# Evaluating the Resources and Environmental Carrying Capacity in Laos Using a Three-Dimensional Tetrahedron Model

**DOI:** 10.3390/ijerph192113816

**Published:** 2022-10-24

**Authors:** Fangyu Zheng, Chiwei Xiao, Zhen You, Zhiming Feng

**Affiliations:** 1 Institute of Geographic Sciences and Natural Resources Research, Chinese Academy of Sciences, Beijing 100101, China; 2 College of Resources and Environment, University of Chinese Academy of Sciences, Beijing 100049, China; 3 Key Laboratory of Carrying Capacity Assessment for Resource and Environment, Ministry of Natural Resources, Beijing 101149, China

**Keywords:** resource and environmental carrying capacity, human settlements, three-dimensional tetrahedron model, comprehensive assessment, Laos

## Abstract

(1) Background: The quantitative evaluation and comprehensive measurement of resources and environmental carrying capacity (RECC) are key links in the study of RECC from classification to synthesis. Laos, as the only landlocked country of Mainland Southeast Asia (MSEA), is an important economic corridor (i.e., China-MSEA Economic Corridor) of the Belt and Road Initiative (BRI). (2) Methods: Based on the human settlements index (HSI), resource carrying index (RCI), and socio-economic development index (SDI), here, a three-dimensional tetrahedron model for the comprehensive assessment of RECC with equilibrium significance was constructed, including HSI-based suitability classification, RCI-based restrictive classification, and SDI-based adaptability classification. Taking provinces as the basic unit, we quantitatively assessed and comprehensively evaluated RECC in Laos using a three-dimensional tetrahedron model. (3) Results: The human settlement environment in Laos is mainly characterized by the moderate suitable category (85%), while the high suitability area (merely 5%) supports more than 30% of the total population. Laos had over 90% of its area in good condition in resources and environmental carrying status (surplus or balanced state), translating into more than 95% of the population. The social and economic development level is mainly characterized by low-level development (43%), with nearly 30% of the population living in these low-level areas. The comprehensive bearing state of resources and environment is characterized by surplus, and 85% of the population is distributed in the surplus area, which occupies 63% of the land. (4) Conclusions: It is possible to better explore the adaptation strategies and countermeasures for enhancing RECC in Laos and provide a scientific reference for regional sustainable development. We believe that the three-dimensional tetrahedron method can be applied to quantitatively evaluate and comprehensively measure RECC at larger scale, e.g., the BRI regions.

## 1. Introduction

Resources and environmental carrying capacity (RECC) are a general term that considers both the resources carrying capacity and environmental carrying capacity as a concept to describe development restrictions. Since it was first proposed in the early 1900s, RECC research has developed over more than a hundred years [1]. It is currently an effective and operational tool to guide regional sustainable development. Global ongoing initiatives, such as the 2030 UN Sustainable Development Goals (SDGs) [2] and the United Nations Collaborative Programme on Reducing Emissions from Deforestation and Forest Degradation in Developing Countries (UN-REDD) [3], have repeatedly emphasized the significance of sustainable development or RECC, especially in tropics. In particular, resources are the basis for human survival, and various human activities will cause changes in the environment, and the two affect each other [4]. In other words, the three aspects of resources, human beings, and the environment are mutually influenced and interdependent [5]. Indeed, human beings have certain limits on the use of resources and the environment’s carrying capacity. This limit identifies the resource carrying capacity and environmental carrying capacity, or RECC [6,7,8]. Along with socio-economic development, utilization and exploitation of resources are indispensable, resulting in some over-exploitation of resources and/or large amounts of waste discharged in the process [9]. The accommodating capacity and self-purification capacity of the resource environment are easily exceeded in the absence of strict controls. This leads to a decrease in productivity and threatens the security of the regional ecosystem [10,11]. Moreover, the destruction of the ecological environment not only restricts the sustainable development of society but also presents an urgent livelihood problem that affects human health [12]. Therefore, sustainable development must be established based on resource carrying capacity and environmental carrying capacity, and it is of great theoretical and practical significance to study the comprehensive assessment of RECC [13,14].

Generally, the research direction of RECC includes the definition of the connotation, the comprehensive determination of the assessment index system, the research and application of comprehensive research methods, and the relationship between man and land [15,16]. At present, the index system in research on comprehensive RECC contains more and more extensive content [17]. According to different bearing objects, researchers set different target layers, or element layers, from the aspects of the economy, society, environment, and resource conditions [18]. Different researchers have different perspectives upon the research on the connotation of the comprehensive RECC [19]. The earliest studies included concepts of various elements of the human geographic environment and land resources [20]. More importantly, RECC mainly resolves the maximum population size (i.e., How many people can Earth support? [21]) that can be accommodated by all our production factors in a certain spatio-temporal domain and under certain conditions of irreversible living and environmental standards [22]. Introducing urban resources into RECC is an inevitable choice in the era of rapid urbanization. It mainly involves the environmental carrying capacity, including land resources [23], atmospheric resources [24], and water resources [25], in addition to comprehensively considering the influence of culture, technology, and systems, and exploring the supporting capacity of a certain resource [26]. Moreover, with the population index as the starting point, RECC is defined as belonging to a specific area. On the premise of maintaining a good ecosystem, we may estimate the maximum number of people that the regional natural environment can accommodate [27].

To date, there are four main types of research on the connotation of RECC [28]. The first type is ecological carrying capacity (i.e., the maximum biomass of individual populations). The second is to characterize the equilibrium state of the load, that is, the “loaded state”. Thirdly, from environmental carrying capacity to ecosystem resilience (i.e., thinking based on the relationship between the population and environment). The last type is integrated within resources and the environment carrying capacity (i.e., thinking based on regional sustainable development), which is the extension and development of carrying capacity, ecological carrying capacity, resource carrying capacity (e.g., land and water), and environmental carrying capacity (or environmental capacity) [29]. Thus, the connotation and methods of RECC are constantly enriched and developed [30]. In particular, a qualitative approach has been developed to delineate RECC at regional scales [31].

In the context of bearing capacity, different research methods exist according to different research subjects, from a single factor to comprehensive factors. These are based on the characteristics of water resource dynamics, randomness, and uncertainty, such as multi-objective optimization [32], artificial neural network modeling [33], fuzzy synthesis modeling [34] and other evaluation methods. Based on the irreplaceability of land resources and the inseparability of the relationship between man and land, some methods can reflect the supply and demand relationship between man and land, such as supply-demand balance modeling [8], ecological footprint and virtual land (or the biologically productive land by the ecological footprint), water footprint and virtual water (or the global flow of virtual water), and energy analysis and virtual energy (or energy equilibrium) [35]. More importantly, the evaluation method of the comprehensive carrying capacity combines the relationship between man and land. It integrates the resource background of soil and water ecology and the regulation of a social economy [36] to obtain the research method for the comprehensive carrying capacity. However, a standard set of evaluation theories and methodologies are still lacking for RECC research to date, leading to much debate over the objectivity and comparability of resultant figures [6]. To this end, starting from the cognition of resource and environmental carrying capacity, we can define the connotation of resource and environmental carrying capacity and integrate the social economy with population, resources, and the ecological environment [37]. Exploring the interaction mechanism between socio-economic and resource and environmental elements, and establishing quantitative relationships between elements, are the problems faced in advancing the research on comprehensive assessment methods of RECC.

This paper defines RECC from three aspects: (1) evaluating the suitability of human settlements, (2) classifying water and soil resources and ecological carrying capacity, (3) and conducting a suitability assessment of social and economic development by combining social and economic factors with population, resources, and the ecological environment [38,39]. How to develop a set of standardized and patterned comprehensive quantitative evaluation methods is the key task and key problem to be solved in the current research on RECC. Here, the mechanism of interactions among human settlement environmental elements, soil and water ecological elements, and social and economic elements is discussed, and a comprehensive evaluation system of the resource carrying capacity under the combined action of multiple elements is established, or the three-dimensional tetrahedron model. This model is judged by the comprehensive carrying index of resources and environment. On this basis, the comprehensive RECC in Laos, as the only landlocked country of Mainland Southeast Asia, is calculated by scale and purpose, and the restrictive factors affecting the comprehensive carrying capacity are analyzed. We believe that our three-dimensional tetrahedron method can be applied to quantitatively evaluate and comprehensively measure RECC at regional scale and provide a scientific basis and useful reference for regional sustainable development.

## 2. Materials and Methods

### 2.1. Study Area

**Figure 1:**
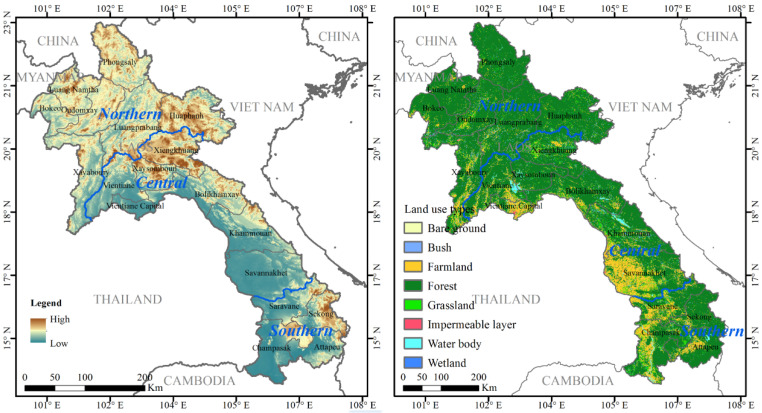
Maps showing the topography and land cover types (2020) in Laos.

The Lao People’s Democratic Republic (Laos) is located in the north of mainland Southeast Asia (Figure 1), with 17 provinces and one municipality (i.e., Vientiane Capital). Generally speaking, it can be divided into three regions: Northern Laos (including seven provinces, namely, Bokeo, Huaphanh, Luang Namtha, Luangprabang, Oudomxay, Phongsaly, and Xayaboury), Central Laos (including Vientiane Capital and six provinces, i.e., Bolikhamxay, Khammouan, Savannakhet, Vientiane, Xiengkhuang, and Xaysomboun) and Southern Laos (including four provinces, namely, Attapeu, Champasak, Sekong, and Saravane). Laos has a tropical and subtropical monsoon climate with abundant rainfall and superior light and heat conditions, which is very suitable for agricultural planting [40]. The main rivers in the area are the Mekong River and its tributaries. Laos is a multi-ethnic, underdeveloped country with a weak industrial foundation and a poor agricultural country. Since the adoption and implementation of the New Economic Mechanism in the 4th Party Congress in 1986, the government of Laos has aimed to eventually curb poverty.

### 2.2. Methods

#### 2.2.1. Human Settlements Index (HSI)

The suitability of regional human settlements can be expressed by the human settlements index (HSI). The HSI is the mathematical synthesis of the normalized relief degree of the land surface (RDLS) (Use ASTER Global Digital Elevation Model (GDEM) data to process extreme and abnormal values, and calculate relative elevation difference, flat ground, and topographic relief (RDLS)), the land cover index (LCI) (The land cover index (LCI) is calculated by using MODIS 2013–2017 multi-year average NDVI and resampled global land cover products), the land surface water abundance index (LSWAI) (The near infrared (NIR) and mid infrared (MIR) data (2013–2017) of MOD13A1 of countries along the line from NASA’s Earth Data platform are used to calculate the surface water index (LSWI), and the surface water abundance and deficiency index (LSWAI) is calculated by combining the precipitation grid products), and the temperature and humidity index (THI) (The monthly average values of national meteorological stations (temperature, relative humidity, precipitation) along the line from the data service room of the National Meteorological Information Center are used for collaborative Kriging interpolation to generate corresponding grid data products, and the temperature humidity index (THI) is calculated). The specific normalization method is as follows:

(1) $$x_{i}^{*}=\frac{x_{i}-\min(X)}{\max(X)-\min(X)},$$

(2) $$x_{i}^{*}=\frac{\max(X)-x_{i}}{\max(X)-\min(X)},$$

$x_{i}^{*}$ is the normalized value of variable x in region i;

$x_{i}$ is the original value of variable x in area i;

X is the set of variables x.

**Figure 2:**
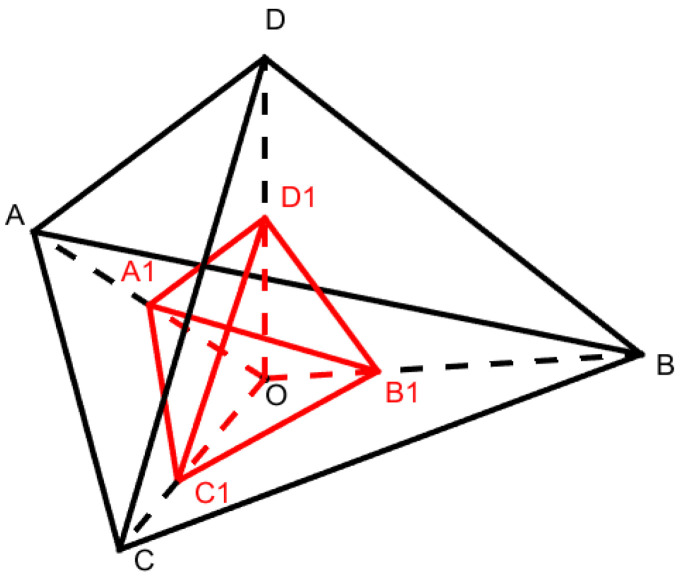
Human settlement environment index model.

In this study, only the RDLS is normalized by Equation (2), while other indices (i.e., LCI, LSWAI, and THI) are normalized according to Equation (1). Upon considering the decisive role of topographical factors on the human settlements, this study established a triangular pyramid model with the RDLS as high and the LCI, the LSWAI, and the THI as the bottom to calculate HSI (Figure 2). In particular, to retain the physical meaning (i.e., equilibrium state), this study carried out the translation processing of the normalized HSI, that is, the mean value of normalization. The calculation method is as follows:

(3) $$\mathrm{HSI}={\mathrm{HSI}}_{\mathrm{one}}-k+1,$$

(4) $${\mathrm{HSI}}_{v}=V_{1}/V_{0\;},$$

(5) $$V_{1}=\frac{\sqrt{3}}{12}\left({\mathrm{OA}}_{1}\times {\mathrm{OB}}_{1}+{\mathrm{OA}}_{1}\times {\mathrm{OC}}_{1}+{\mathrm{OB}}_{1}\times {\mathrm{OC}}_{1}\right)\times {\mathrm{OD}}_{1\;},$$

(6) $$V_{0}=\frac{\sqrt{3}}{12}\left(\mathrm{OA}\times \mathrm{OB}+\mathrm{OA}\times \mathrm{OC}+\mathrm{OB}\times \mathrm{OC}\right)\times \mathrm{OD},$$

HSI is the mean normalization of human settlements index.

HSI_one_ is the HSI normalized by HSI_v_;

k is the mean value of HSI_one_ in the study area;

V_1_ is the volume of the tetrahedron A_1_B_1_C_1_D_1_;

V_0_ is the volume of the tetrahedron ABCD;

OA_1_, OB_1_, OC_1_, and OD_1_ are the actual values normalized by the LCI, LSWAI, THI, and RDLS, respectively; OA, OB, OC, and OD are the normalized standard values of LCI, LSWAI, THI, and RDLS, all of which are equal to one.

#### 2.2.2. Resource Carrying Index (RCI) Model

The limitation of regional water and soil resources and the ecological environment can be characterized by the resource carrying index (RCI). The RCI is a mathematical synthesis of the land carrying index (LCI) (The data come from the National Basic Condition Platform for Science and Technology—National Earth System Science Data Center), water carrying index (WCI) (Precipitation data come from MSWEP v2 precipitation dataset [41]), and ecological carrying index (ECI) (Food and Agriculture Organization of the United Nations [42]). It reflects the comprehensive bearing status of regional water and soil resources and the ecological environment.

**Figure 3:**
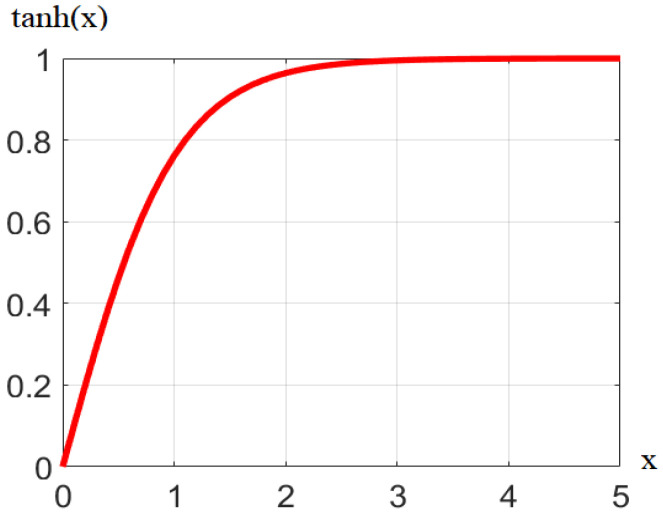
Schematic diagram of the hyperbolic tangent function.

To eliminate the excessive surplus or deficit of a certain type of resource-bearing state in the region during exponential fusion, the information coverage of other types of resource-bearing states is covered. Here, the hyperbolic tangent function (tanh) is used to normalize the reciprocal of each load-bearing index (Figure 3) and retains the actual physical meaning of the equilibrium state when the load-bearing index is one. In addition, based on the three stages of the international mainstream urbanization process [28] (Table 1), this paper assigns different weights to the three carrying indexes (i.e., LCI, WCI, and ECI) in regions with different urbanization stages and combined with the actual situation. The specific calculation method of RCI is as follows:

(7) $$\mathrm{RCI}=W_{L}\times {\mathrm{LCI}}_{t}+W_{W}\times {\mathrm{WCI}}_{t}+W_{E}\times {\mathrm{ECI}}_{t},$$

(8) $${\mathrm{LCI}}_{t}=\tan h(\frac{1}{\mathrm{LCI}})-\tan h(1)+1,$$

(9) $${\mathrm{WCI}}_{t}=\tan h(\frac{1}{\mathrm{WCI}})-\tan h\left(1\right)+1,$$

(10) $${\mathrm{ECI}}_{t}=\tan h(\frac{1}{\mathrm{ECI}})-\tan h\left(1\right)+1,$$

LCI is the land resource carrying index;

WCI is the water carrying index;

ECI is the ecological carrying index;

LCI_t_ is the LCI after hyperbolic tangent translation;

WCI_t_ is the WCI after hyperbolic tangent translation;

ECI_t_ is the ECI after hyperbolic tangent translation;

RCI is the resource carrying index of the weighted capital of soil and water ecological carrying index.

#### 2.2.3. Socio-Economic Development Index (SDI) Model

**Figure 4:**
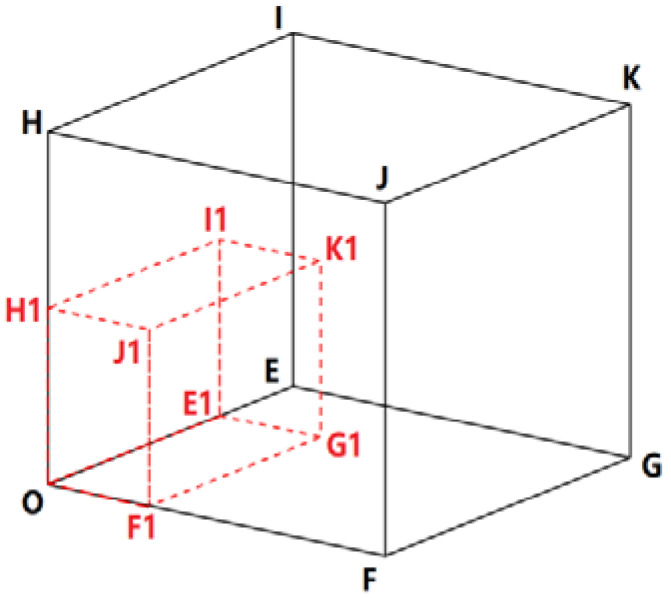
Schematic diagram of socio-economic development index (SDI) model.

Regional socio-economic adaptation can be characterized by the SDI. The SDI is a mathematical synthesis of the human development index (HDI) (United Nations Development Programme), the transport accessibility index (TAI) (United Nations Development Programme), and the urbanization index (UI) (United Nations Development Programme). This study established a three-dimensional cube model to fuse the normalized HDI, TAI, and UI to reduce the coverage of each sub-indicator to the extreme values of other indicators and then normalizes the mean (Figure 4). Its specific calculation formula is as follows:

(11) $$\mathrm{SDI}={\mathrm{SDI}}_{\mathrm{one}}-k+1,$$

(12) $${\mathrm{SDI}}_{v}=V_{1}/V_{0},$$

(13) $$V_{1}={\mathrm{OE}}_{1}\times {\mathrm{OF}}_{1}\times {\mathrm{OH}}_{1},$$

(14) $$V_{0}=\mathrm{OE}\times \mathrm{OF}\times \mathrm{OH},$$

SDI is the mean normalization of the socio-economic development index.

SDI_one_ is the SDI normalized by SDI_v_;

k is the mean value of SDI_one_ in the study area;

V_1_ is the volume of the red cube (i.e., OE_1_F_1_G_1_H_1_I_1_J_1_K_1_);

V_0_ is the volume of the black cube (i.e., OEFGHIJK);

OE_1_, OF_1_, and OH_1_ are the normalized, actual values of HDI, TAI, and UI, respectively;

OE, OF, and OH are the normalized standard values of HDI, TAI, and UI, all equal to one.

#### 2.2.4. Resource and Environment Comprehensive Carrying Capacity Index (RECCI) Model

**Figure 5:**
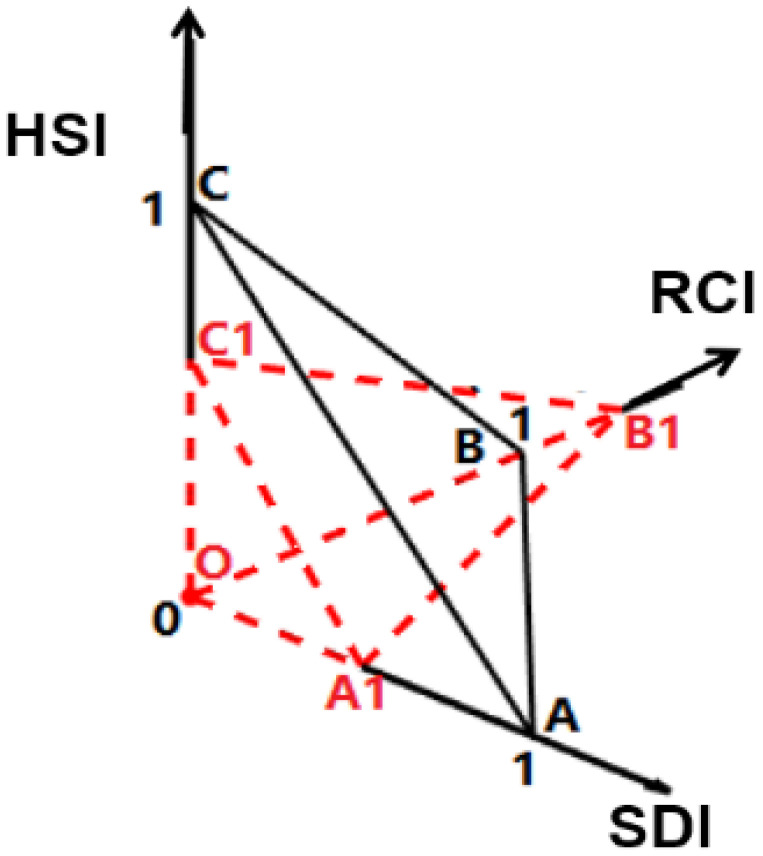
Schematic diagram of the resource environment comprehensive carrying capacity index (RECCI) model. HSI, human settlements index; RCI, resource carrying index; SDI, socio-economic development index.

On the basis of keeping the comprehensive index as one (equilibrium state), this paper used a three-dimensional tetrahedron model to calculate the RECCI (Figure 5). Its specific calculation formula is as follows:

(15) $$\mathrm{RECCCI}=V_{1}/V_{0},$$

(16) $$V_{1}=\frac{1}{6}\left({\mathrm{OA}}_{1}\times {\mathrm{OB}}_{1}\times {\mathrm{OC}}_{1}\right),$$

(17) $$V_{0}=\frac{1}{6}\left(\mathrm{OA}\times \mathrm{OB}\times \mathrm{OC}\right),$$

RECCI is the comprehensive carrying index of resources and environment;

V_1_ is the volume of the red tetrahedron (i.e., OA_1_B_1_C_1_);

V_0_ is the volume of the black tetrahedron (i.e., OABC);

OA_1_, OB_1_, OC_1_ are the actual values of SDI, RCI, and HSI, respectively;

OA, OB, and OC are the standard balance values of SDI, RCI, and HSI, respectively, all of which are equal to one.

When RECCI = 1, it represents the theoretical balance state of regional resources and environment. According to the PREDI index, the comprehensive RECC can be divided into the following three warning levels: (1) when RECCI < 0.875, it represents that the carrying capacity of resources and environment is in an overloaded state, and the development space needs to be expanded; (2) When RECCI is between 0.875 and 1.125, it means that the carrying capacity of resources and environment is in balance and needs to be adjusted appropriately; (3) When RECCI > 1.125, it means that the carrying capacity of resources and environment is in surplus and there is still room for development.

### 2.3. Data Sources

In this study, (1) 30-m Advanced Spaceborne Thermal Emission and Reflection Radiometer (ASTER)-Global Digital Elevation Model (GDEM) data (Version 2) were used as the elevation data (or RDLS). (2) The land cover data (or LCI) were extracted from the Global 30-m Land cover during 2015, the overall accuracy of GlobeLand30 V2010 data is 83.50%, and the Kappa coefficient is 0.78. (3) The LSWAI data were obtained from the NASA Earth Data Platform. (4) The climate element of human settlements (i.e., THI) came mainly from the National Meteorological Information Center of China.

## 3. Results

### 3.1. HSI-Based Suitability Classification

**Figure 6:**
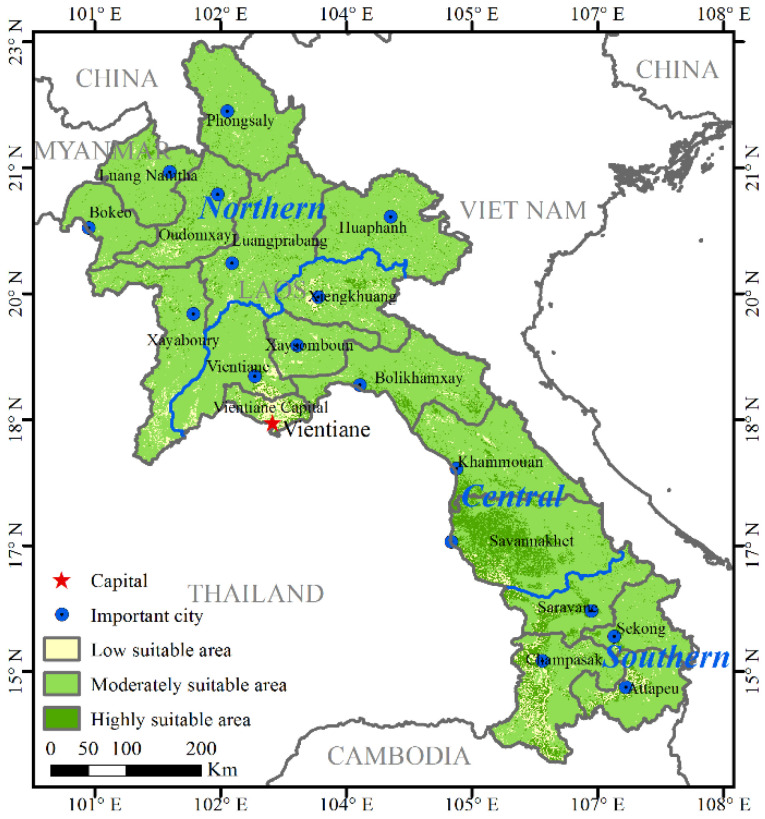
Spatial pattern of the natural suitability (or three sub-categories) of human settlements using the HSI in Laos.

The HSI-based suitability assessment of human settlements in Laos indicated that (Table 2 and Figure 6), the state of human settlements is mainly characterized by the area’s suitability, and the suitable types of human settlements cover almost the entire territory of Laos.

The high suitability area (merely 5%) of human settlements supports more than 30% of the total population, and it is concentrated in the Savannakhet Plain in southern Laos (Figure 6). Farmland and forests account for a large proportion of these areas, which are basically not restricted by hydrological, climatic, and ground cover conditions. The areas in northern, central, and southern Laos with high suitability areas of human settlements accounted for 23.02%, 44.44%, and 32.54%, respectively, and the corresponding population proportions were 9.69%, 58.64%, and 31.67%, respectively.

Next, the moderate suitability area of human settlements in Laos is 201,900 km^2^, accounting for 85.29% of the area and about 50% of the population. The terrain in this area is mostly hills and plateaus, such as the Xiengkhuang Plateau. The moderate suitability area for human settlement in the northern, central, and southern regions of Laos account for 46.33%, 37.02%, and 16.65% (Table 2), respectively, and the corresponding population accounts for 51.02%, 37.23%, and 11.57%.

Also, the area of Laos with a low suitability area for human settlements is 22,300 km^2^, accounting for 5.30% of the total. These areas are distributed across 18 provinces and/or cities, with relatively high concentrations in Vientiane, Xiengkhouang, and Champasak. The corresponding population in the low suitability areas of human settlements is 1.29 million, accounting for 20.59% of the total population. These regions have low average annual rainfall, high average annual temperature, high humidity, and an overall hot climate. However, due to the relatively developed social economy, the population density is relatively large at 58 people/km^2^. The low suitability areas of human settlements in the northern, central, and southern regions of Laos account for 13.90%, 56.95%, and 29.15%, respectively, and the corresponding population proportions are 9.17%, 65.07%, and 25.75% (Table 2).

### 3.2. RCI-Based Restrictive Classification

**Figure 7:**
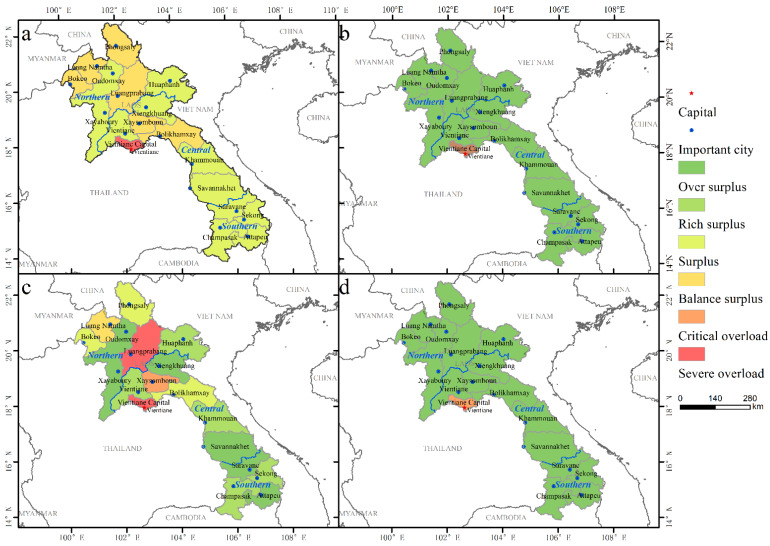
Spatial pattern of the (**a**) resource and environment restrictive classification, (**b**) ecological carrying status, (**c**) land resource bearing status, and (**d**) water resource bearing status in Laos.

The RCI-based evaluation of resource carrying limitation in Laos indicated that, the resource carrying capacity of Laos is mainly characterized by surplus, and 17 provinces are in a state of surplus or above resource carrying capacity. The area accounts for over 90%, and the corresponding population accounts for 90% (Figure 7 and Table 3).

The surplus area of resource carrying capacity in Laos includes 11 provinces within an area of about 163,000 km^2^, accounting for 68.83% of the total. These areas are concentrated mostly in southern Laos and also in the northern part (especially Luangprabang and Phongsaly). Two-thirds of the Laotian population live in surplus areas, characterized by a population of 4347 million and a population density of 27 people/km^2^. The natural conditions in this area are relatively favorable, with a relatively high proportion of lowlands, which are, for the most part, not limited by land resources, water resources, and ecological resources.

The areas with balance surplus resource carrying capacity in Laos include six provinces, with an area of about 69,900 km^2^, accounting for 29.52% of the total area, mainly in northern and central Laos. The main characteristic of these areas is the relatively low land carrying capacity. Such areas can be further divided into two categories. The first is the counties with low carrying capacity and actual population, such as Phongsaly, Luang Namtha, Bokeo, and Xaysomboun. The other category is counties with relatively high carrying capacity and actual population, such as Luangprabang and Bolikhamxay.

Furthermore, there is one province in Laos with an overloaded resource carrying capacity, with the Vientiane Capital in the center, constituting an area of about 55,600 km^2^, accounting for 0.39% of the total. As the most urbanized area in Laos, the ecological and water resources carrying capacity of the Vientiane Capital are its main limiting factors.

### 3.3. SDI-Based Adaptability Classification

**Figure 8:**
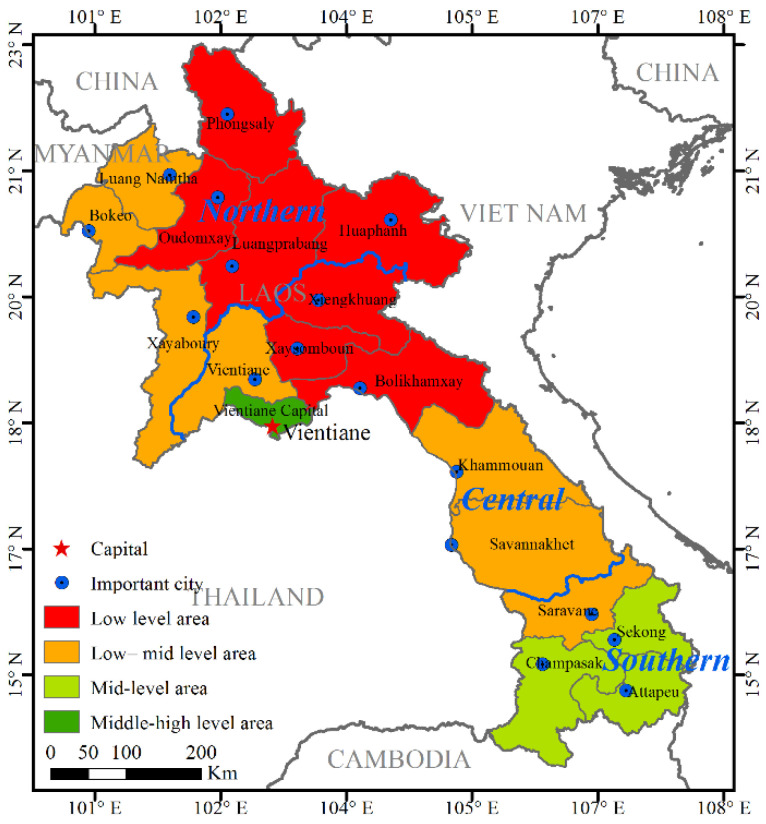
Spatial pattern of classification of socio-economic development in Laos.

The evaluation results of the socio-economic adaptability of Laos based on SDI show (Table 4 and Figure 8) that most areas belong to low-level development areas, which limits the comprehensive RECC.

The high level of socio-economic development in Laos exists only in the Vientiane capital (Figure 8), and the population density and economic density are 10 times and 1.8 times the national average, respectively. The levels of human development, traffic accessibility, and urbanization in this region are in relative balance.

The middle-level of socio-economic development in Laos includes the three southern provinces of Sekong, Attapeu, and Champasak, spanning about 33,000 km^2^ and accounting for 13.95% of the total area. The population of these areas is 946,000, accounting for 14.57% of the total, and the population density is 29 people/km^2^, which is mainly limited by the level of urbanization.

The low-level socio-economic areas of Laos include seven provinces (Table 4), with an area of about 98,100 km^2^, accounting for 41.44% of the total, mainly in northern and central Laos. The population of these areas is close to three million, accounting for 44.89% of the total, and the population density is 30 people/km^2^. The areas of low and medium socio-economic levels in northern, central, and southern Laos collectively account for about 1/3 of their respective regions, and the corresponding population proportions are 25.26%, 27.83%, and 46.91%, respectively. The level of urbanization in these regions is less than two thirds of the national average.

The low levels of socio-economic development in Laos include seven provinces, with an area of about 101,700 km^2^, accounting for 42.97% of the total. These are mainly distributed in northern Laos. The population of such areas is 1.81 million, accounting for 27.90%, and the population density is 18 people/km^2^. Regarding the main influencing factors of each province, the provinces of Oudomxay, Xaysomboun, and Bolikhamxay are mainly limited by traffic levels, and the normalized traffic accessibility index is less than one-tenth of the national average. At the same time, the provinces of Huaphanh, Xiengkhuang, Luangprabang, and Phongsaly are mainly limited by their urbanization level. Here, the average normalized urbanization index is less than a quarter of the national average.

### 3.4. RECCI-Based Warning Rating

**Figure 9:**
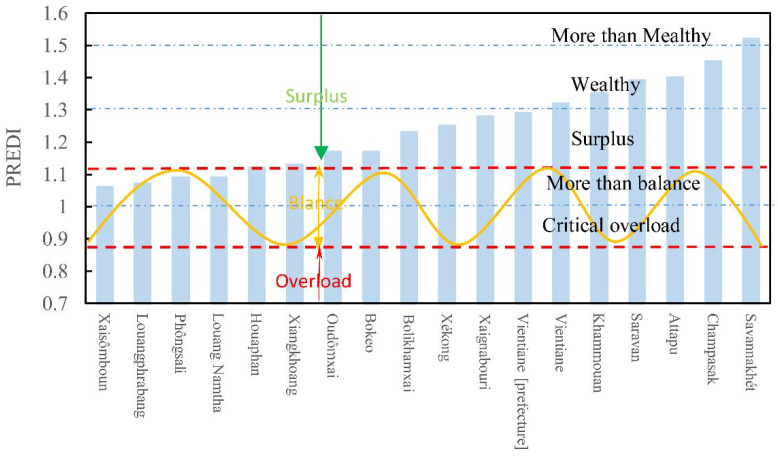
Grading of RECCI of resources and environment by province in Laos.

**Figure 10:**
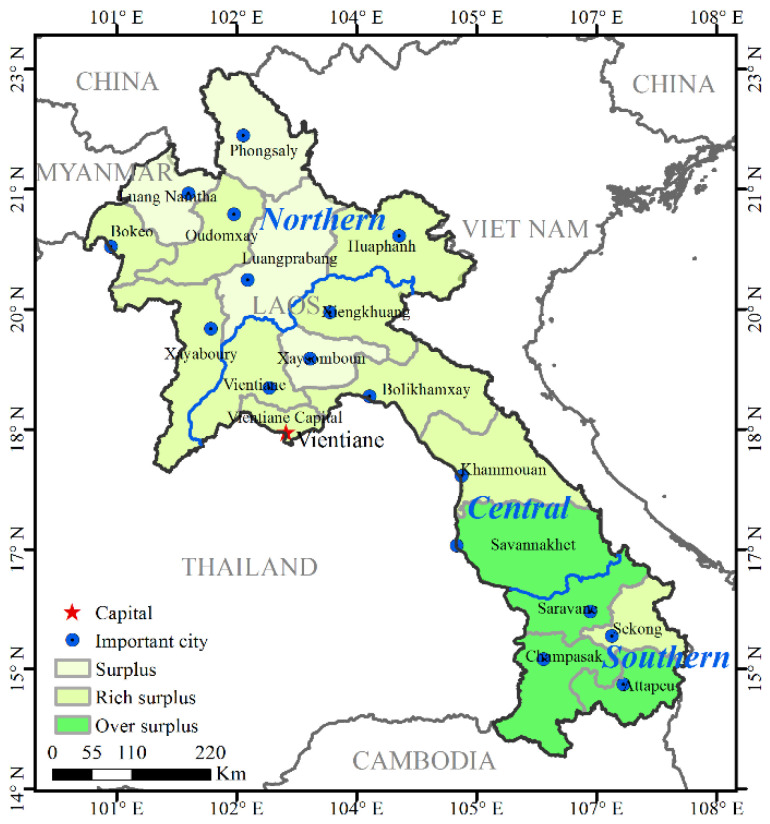
Spatial distribution of resource environment comprehensive carrying status in Laos.

According to Figure 9, the RECCI of all provinces in Laos is greater than one, which is a surplus state, and the index is higher in the southern part and lower in the northern region. There are 4, 8, and 4 provinces in Laos with surplus, rich, and surplus-rich resources, respectively (Figure 10). In terms of sub-indices, the overall level of social and economic development in Laos is relatively low. Except for the social and economic index of Vientiane capital, which is 1.16, the corresponding indices of the other provinces are between 0.98 and 1.02, and the index difference is small. The regional differences in resource endowment and natural conditions are obvious. The HSI of Laotian provinces ranges from 0.93 to 1.23, and the RCI ranges from 0.95 to 1.23.

The provinces in Laos with an over surplus state of comprehensive resource and environmental carrying status include Saravane, Attapeu, and Champasak, all of which are located in the southern plains. The area of this region is about 57,600 km^2^, accounting for 1/4 of the country, and the corresponding population is about 2.20 million, accounting for 1/3 of the total population. The average value of the regional comprehensive carrying index of resources and environment is 1.44 times the national average, and there is room for the development of resources and environment.

As indicated in Table 5, the HSI of the over surplus areas is 1.17 times that of the national level. The average resource and environmental carrying index are 1.22 times the national average. The average socio-economic suitability index was 1.01 times the national average (Table 5). On the basis of superior resource endowment, the natural suitability of the high suitability area of human settlements and the relatively high level of social and economic development have jointly improved the comprehensive carrying capacity of regional resources and the environment. Among them, Attapeu province, which is located on the southeastern border of Laos, is worthy of attention. It is located in the center of the basin in the downstream area of the Mekong River and has superior resource endowments. Its RECCI is as high as 1.40, but the population density is less than half of the national average population density and only 1/3 of the average density of a surplus-rich area.

There are 10 provinces in Laos that have a rich surplus in comprehensive resources and the environment, mainly located in the northern and central regions, with an area of about 130,200 km^2^, accounting for half of the country’s land area. The average value of the regional RECCI is 1.23, which is comparable to the national average (1.24), and there is room for the development of resources and the environment. Overall, the average HSI in wealthy areas is 1.03 times the national average. The average RECCI is 1.18 times the national average. The average socio-economic adaptation index was 1.01 times the national average (Table 5), slightly higher than the national average. Specifically, Vientiane capital has the lowest resource carrying capacity, but the human settlement environment and social economy rank first, collectively enhancing its comprehensive RECC.

On the contrary, Huaphanh, Xiengkhuang, and Oudomxay have strong resource carrying capacity, but poor human environment foundation and social and economic development have jointly affected their comprehensive RECC. In the remaining six provinces, the resource carrying capacity, suitability of the living environment, and social and economic adaptability are all at upper-middle levels. The comprehensive carrying status of resources and environment is good, and there is room for the development of resources and environment.

There are four provinces in Laos with both a resource and environmental surplus, i.e., Attapeu, Champasak, Saravane, and Savannakhet. These are mainly located in the northern region. The average value of the RECCI in this type of district is 1.08, which is lower than the national average (1.24), and the room of development of resources and environment is limited. In particular, the average RECCI of such regions is about 95% of the national average. The average socio-economic adaptation index is about 98% of the national average. The average human settlement index is about 92% of the national average (Table 5), all of which is generally lower than the national average. It can be seen that the resource carrying capacity of this type of area is not strong, and the foundation of human settlements and social and economic development further limits the exertion of its RECC. Notably, Luangprabang, the ancient capital in the north, has the largest population size and population density. The population accounts for half of such areas, and the population density is 26 people/km^2^, close to the national average. Due to the limitation of land resources, the resource carrying capacity here is low, but the province is rich in water resources, noting that it is particularly important to improve the allocation efficiency of regional water and soil resources.

## 4. Discussion

The comprehensive assessment of resource and environmental carrying capacity (RECC) shows that the resource-carrying status in Laos is relatively good, and the overall levels are in surplus (the value of RECCI is 1.25). The resource-carrying capacity is much higher than the actual population, and there is a lot of room for the development in Laos. From the perspective of water resources, land resources, and ecological carrying status, Laos has abundant water resources and a good ecological background. The carrying capacity of land resources is relatively weak, and the shortage or unbalanced matching of land resources has become the main reason for the low carrying capacity of land resources in some regions. Although the population of Laos has not exceeded the carrying capacity of soil and water resources, some cities are still in a state of critical overloads, such as Luangprabang and Vientiane capital. Therefore, to fundamentally reduce the land resource restrictions on the population distribution in Laos, it is necessary to appropriately guide the orderly flow of population from the land resource overloaded area to the land resource surplus area. Promoting the coordination of population distribution and resource carrying capacity in different regions of Laos will reduce the restriction of land resources on Laos’ RECC.

The natural suitability of human settlements in Laos is high (suitable area up to 95%), even if topographical conditions represent the limiting factor restricting the suitability of human settlements. However, the renewability and man-made regulation of hydrology and ground cover increase the suitability of the overall human settlement environment in Laos, and the human settlement environment does not constitute a constraining factor on the comprehensive RECC. On the other hand, the low level of urbanization and lack of transportation infrastructure have reduced the comprehensive RECC in Laos (80% of areas are at a low level of social and economic development). Therefore, to continuously improve the comprehensive carrying capacity of national resources and the environment, it is necessary to comprehensively solve the problem of social and economic adaptability and speed up external and/or international investments in social development. It is also necessary to comprehensively improve national social and economic development levels and actively strive for international capital. In the future, carrying out assistance and cooperation with neighboring countries (such as China and Vietnam) will speed up the construction of infrastructure (such as the China–Laos railway) and change the “land-locked country” into a “land-linked country”.

For areas such as Luangprabang, Phongsaly, Luang Namtha, and Xaysomboun, which have a slight surplus and risk of overloading, the comprehensive carrying index (i.e., RECCI) of resources and environment in these regions is lower than the national level (average value is 1.25). Except for Luangprabang, the HSI, RCI, and SDI of other provinces are all at low levels. Therefore, to improve the comprehensive RECC in such areas, a three-pronged approach is necessary. First, according to the suitability of human settlements, guide the population to migrate in an orderly manner to areas with a high degree of suitability. The second is to focus on improving the carrying capacity of land resources, giving full consideration to the natural advantages of the abundant Laotian water resources, and improving the allocation and utilization efficiency of water and soil resources. Engineering (e.g., build reservoirs) and biological (e.g., forbid swidden agriculture) measures improve land productivity and reduce the degree of limitation of land resources on comprehensive RECC. The third prong focuses on solving the problems of survival and development of the population in poverty-stricken areas. Most of these areas are rural, so agricultural and rural reforms should be implemented, and the transfer of surplus agricultural and rural labor to urbanized areas should be encouraged in an orderly manner.

Provinces and regions with rich RECC of Laos are mainly divided into two regional types. The first type represents areas with a strong resource carrying capacity, where lacking socio-economic development affects its potential (SDI < 1), such as in Huaphanh (0.98), Xiengkhuang (0.99), and Oudomxay (0.99). In this area, the advantages of natural resources (e.g., water and land resources) should be further exerted, the investment in agricultural science and technology should be increased, and the construction of water conservancy facilities should be strengthened. It is necessary to fully tap the potential of agricultural production (i.e., multiple cropping system), develop and construct national-level grain reserve bases, increase the ability to reserve and control grain and other materials, and ensure national food security. Meanwhile, strengthening the construction of transportation infrastructure and guiding the population to gather in villages and towns on flat land with a high degree of suitability is favorable for the living environment. The second type represents areas obviously affected by resource carrying capacity (less than 1.0), such as Vientiane capital (0.95). Such urbanized areas are limited by their own, and it is difficult for land resources and water resources to maximize its value. In other words, the unique features of a local environment always give special characteristics to its inhabitants. It is necessary to meet the needs of population growth for resources through the cross-regional occupation of resources and cross-regional allocation of materials. Such areas should strengthen their intensive land use, improve the comprehensive production capacity of cultivated land resources, and finally solve the problem through regional planning.

For provinces with abundant RECC, such as Savannakhet and other areas of the southern plains, meanwhile, the living environment and social economy are at good levels, and there is a lot of room for population development. The dominant positioning of this type of area is the production and living functional area. To ensure agricultural production, the focus should be placed on optimizing population function and industrial agglomeration. In addition, strengthening infrastructure construction, actively introducing foreign capital, and further exploring and identifying the advantageous conditions of each region would prove helpful. Absorbing the population transferred from agriculture to the maximum extent will promote the development and agglomeration of suitable industries in various regions and form a developmental pattern of complementary advantages and benign interaction.

## 5. Conclusions

This paper is based on the classification evaluation of water resources, land resources, and ecological environment carrying capacity. It combines the evaluation of the suitability of human settlements with the evaluation of social and economic development adaptability. This study puts forward research ideas and technical routes to comprehensively evaluate the resource and environmental carrying capacity (RECC) of a “suitability zoning, restrictive classification, adaptive classification, and warning classification”. A three-dimensional tetrahedron model for the comprehensive evaluation of RECC with equilibrium significance is constructed, providing technical support for realizing a comprehensive evaluation of the RECC for Laos.

The essence of RECC is to answer the question of the coordinated development of the “human-land” relationship. As a “ruler” to measure the coordinated development of population, resources, and environment, human activities must be kept within the limits of the resources, ecology, and environment that the Earth can bear. Among them, the suitable zoning of human settlements represents the premise of the evaluation of RECC. The classification of resource limitations represents the basis for research on the RECC. The classification of social and economic adaptability is the difference between the suitability of human settlements and the limitations of resources and the environment. On this basis, it is important to further consider the impact or response of social and economic development on resources and the environment. Therefore, to improve the regional RECC (e.g., Laos), it is necessary to comprehensively resolve problems related to resources and environmental limitations and social and economic adaptability. We must further pay attention to the coordinated development of the population, resources, environment, society, and economy in backward provinces.

## Figures and Tables

**Table 1:** Weights of three carrying indexes Stage of urbanization process.

	Proportion of Urban Population	W_L_	W_W_	W_E_
Initial stage	[0,30)	0.5	0.3	0.2
Mid stage	[30,70)	1/3	1/3	1/3
Later stage	[70,100)	0.2	0.5	0.3

**Table 2:** Results of the natural suitability assessment of human settlements using the HSI in Laos.

Types	Region	Land		Population		
		Area (10^4^ km^2^)	Proportion (%)	Quantity (10^4^)	Proportion (%)	Density (People/km^2^)
High suitability area	Northern	0.29	23.02	19.4	9.69	67
	Central	0.56	44.44	117.44	58.64	210
	Southern	0.41	32.54	63.42	31.67	155
	Sub-total	1.26	5.32	200.26	30.85	159
Moderate suitability area	Northern	9.35	46.33	163.01	51.02	17
	Central	7.47	37.02	118.94	37.23	16
	Southern	3.36	16.65	37.54	11.75	11
	Sub-total	20.18	85.26	319.49	49.22	16
Low suitability area	Northern	0.31	13.90	11.87	9.17	38
	Central	1.27	56.95	84.22	65.07	66
	Southern	0.65	29.15	33.33	25.75	51
	Sub-total	2.23	9.42	129.42	19.94	58

**Table 3:** Statistics of different resource carrying state regions in Laos.

Types	Region	Province		Land		People		
		Quantity	Proportion (%)	Area (10^4^ km^2^)	Proportion (%)	Quantity (10^4^)	Proportion (%)	Density (People/km^2^)
Surplus	Northern	4	36.36	6.34	38.90	122.3	28.13	19
	Central	2	18.18	3.45	21.17	81.1	18.66	24
	Southern	5	45.45	6.51	39.94	231.3	53.21	36
	Sub-total	11	61.11	16.3	68.83	434.7	66.96	27
Balance surplus	Northern	4	66.67	4.81	68.81	96.5	72.89	20
	Central	2	33.33	2.17	31.04	35.9	27.11	17
	Southern	-	-	-	-	-	-	-
	Sub-total	6	33.33	6.99	29.52	132.4	20.39	19
Overload	Northern	-	-	-	-	-	-	-
	Central	1	5.56	0.39	1.65	82.1	12.65	211
	Southern	-	-	-	-	-	-	-
	Sub-total	1	5.56	0.39	1.65	82.1	12.65	211

**Table 4:** Statistics of different socio-economic development levels regions in Laos.

Types	Region	Province		Land		People		
		Quantity	Proportion (%)	Area (10^4^ km^2^)	Proportion (%)	Quantity (10^4^)	Proportion (%)	Density (People/km^2^)
Middle-high- level area	Northern	-	-	-	-	-	-	-
	Central	1	100	0.39	100.00	82.1	100.00	212
	Southern	-	-	-	-	-	-	-
	Sub-total	1	5.56	0.39	1.64	82.1	12.65	212
Mid-level area	Northern	-	-	-	-	-	-	-
	Central	-	-	-	-	-	-	-
	Southern	3	100.00	3.30	100.00	94.6	100.00	29
	Sub-total	3	16.67	3.30	13.95	94.6	14.57	29
Low-mid- level area	Northern	3	42.86	3.16	32.16	73.6	25.26	23
	Central	2	28.57	3.45	35.12	81.1	27.83	24
	Southern	2	28.57	3.21	32.72	136.7	46.91	43
	Sub-total	7	38.89	9.81	41.44	291.4	44.89	30
Low-level area	Northern	5	71.43	8.00	78.64	145.2	80.18	18
	Central	2	28.57	2.17	21.36	35.9	19.82	17
	Southern	-	-	-	-	-	-	-
	Sub-total	7	38.89	10.17	42.97	181.1	27.90	18

**Table 5:** Comprehensive bearing status of resources and environment in Laos.

Types	Province	Land			People		RECCI	HSI	SDI	RCI
		Area	Proportion	Quantity	Proportion	Density				
		(10^4^ km^2^)	(%)	(10^4^)	(%)	(People/km^2^)				
Surplus	Xayaboury	0.71	3	8.5	1.31	12	1.06	0.96	0.99	1.1
	Luangprabang	1.69	7.13	43.2	6.65	26	1.07	0.98	0.99	1.08
	Phongsaly	1.63	6.87	17.8	2.74	11	1.09	0.96	0.99	1.15
	Luang Namtha	0.93	3.94	17.6	2.71	19	1.09	0.98	1	1.13
	Sub-total	4.9	20.71	87.1	13.42	18	1.08	0.97	0.99	1.12
Rich surplus	Huaphanh	1.65	6.97	28.9	4.45	18	1.12	0.94	0.98	1.22
	Xiengkhuang	1.59	6.71	24.5	3.77	15	1.13	0.93	0.99	1.22
	Oudomxay	1.54	6.49	30.8	4.74	20	1.17	0.98	0.99	1.23
	Bokeo	0.62	2.62	17.9	2.76	29	1.17	1.01	1.01	1.16
	Bolikhamxay	1.49	6.28	27.4	4.22	18	1.23	1.06	1	1.15
	Sekong	0.77	3.24	11.3	1.74	15	1.25	1.02	1	1.19
	Xayaboury	1.64	6.92	38.1	5.87	23	1.28	1.03	1	1.23
	Vientiane	0.39	1.66	82.1	12.65	209	1.29	1.17	1.16	0.95
	Vientiane Capital	1.59	6.73	41.9	6.45	26	1.32	1.09	1	1.22
	Khammouan	1.63	6.89	39.2	6.04	24	1.35	1.11	1	1.21
	Sub-total	12.9	54.49	342.1	52.7	27	1.23	1.03	1.01	1.18
Over surplus	Saravane	1.07	4.51	39.7	6.12	37	1.39	1.16	1	1.23
	Attapeu	1.03	4.36	13.9	2.14	13	1.4	1.12	1.02	1.22
	Champasak	1.54	6.51	69.4	10.69	45	1.45	1.16	1.01	1.21
	Savannakhet	2.18	9.2	97	14.94	45	1.52	1.23	1	1.23
	Sub-total	5.82	24.58	220	33.89	38	1.44	1.17	1.01	1.22

## Data Availability

Some or all data and models that support the findings of this study are available from the corresponding author upon reasonable request.
